# Using Pd-Doped γ-Graphyne to Detect Dissolved Gases in Transformer Oil: A Density Functional Theory Investigation

**DOI:** 10.3390/nano9101490

**Published:** 2019-10-19

**Authors:** Xiaoxing Zhang, Rongxing Fang, Dachang Chen, Guozhi Zhang

**Affiliations:** 1 Hubei Key Laboratory for High-efficiency Utilization of Solar Energy and Operation Control of Energy Storage System, Hubei University of Technology, Wuhan 430068, China; xiaoxingzhang@whu.edu.cn (X.Z.); 101900155@hbut.edu.cn (R.F.); 20191062@hbut.edu.cn (G.Z.); 2 School of Electrical Engineering and Automation, Wuhan University, Wuhan 400044, China; 3 State Key Laboratory of Power Transmission Equipment & System Security and New Technology, Chongqing University, Chongqing 400044, China

**Keywords:** Pd-Doped graphyne, dissolved gases, adsorption, density functional theory (DFT)

## Abstract

To realize a high response and high selectivity gas sensor for the detection dissolved gases in transformer oil, in this study, the adsorption of four kinds of gases (H_2_, CO, C_2_H_2_, and CH_4_) on Pd-graphyne was investigated, and the gas sensing properties were evaluated. The energetically-favorable structure of Pd-Doped γ-graphyne was first studied, including through a comparison of different adsorption sites and a discussion of the electronic properties. Then, the adsorption of these four molecules on Pd-graphyne was explored. The adsorption structure, adsorption energy, electron transfer, electron density distribution, band structure, and density of states were calculated and analyzed. The results show that Pd prefers to be adsorbed on the middle of three C≡C bonds, and that the band gap of γ-graphyne becomes smaller after adsorption. The CO adsorption exhibits the largest adsorption energy and electron transfer, and effects an obvious change to the structure and electronic properties to Pd-graphyne. Because of the conductance decrease after adsorption of CO and the acceptable recovery time at high temperatures, Pd-graphyne is a promising gas sensing material with which to detect CO with high selectivity. This work offers theoretical support for the design of a nanomaterial-based gas sensor using a novel structure for industrial applications.

## 1. Introduction

At present, 2D materials are attracting much attention after the successful synthesis of graphene by micromechanical exfoliation in 2004 [1]. Because of the excellent physical and chemical properties of graphene, including high surface area and activity, high thermal conductivity, good heat dissipation, sensitive light response, and gas/ion response, graphene has found broad application value and development space [2,3,4]. Nonetheless, the difficulty of operating the zero value of the band gap restricts its wide use in industrial processes. Based on this, several emerging 2D materials have been exploited, including transition metal dichalcogenides (TMDs) [5,6], isomers of graphene [7], metal carbides and nitrides [8], 2D metal-organic frameworks [9], etc. Because of their more flexible band structures, these materials could have more promising applications in the field of optical and electronic devices.

Among numerous 2D carbon allotropies, graphyne (GY) remains one of the most popular monolayer materials [10,11,12]. The two most popular structures, γ-graphyne (γ-GY) and graphdiyne (GDY), both have sp^2^- and sp-hybridized carbon atoms. The difference is that in γ-GY, only one C-C triple bond is present between two hexagonal carbon rings, while two triple bonds occur between the two rings in GDY [10,11,12]. Previous studies using theoretical methods have shown that several structures of graphyne are physically and chemically stable, such as α-graphyne (α-GY), β-graphyne (β-GY), 6,6,12-graphye [11], etc. In contrast to graphene and graphite, C≡C triple bonds appear in all types of graphyne. The ratio of bond type of the acetylenic linkages of α-GY, β-GY, and γ-GY is different. α-GY has 100% acetylenic linkages, while β-GY and γ-GY have 66.67% and 33.33%, respectively [13]. Different types of GY also have different band structures. For α-GY and β-GY, the valence band and conduction band cross to one point, while γ-GY has a direct band gap of about 0.471 eV, as measured using the generalized gradient approximation (GGA) approach [14]. But graphdiyne (GDY) was the first one reported to be synthesized in experiment [15]. However, although γ-GY has not been prepared practically, several theoretical studies have shown that it has promising uses in field of catalysis, energy storage, gas/ion sensor, and electronic/optical devices. γ-GY may be a promising catalyst for oxygen reduction reactions (ORRs), nitrogen reduction reactions (NRRs) etc. After the doping of metal or non-metal atoms, graphyne has excellent catalytic performance, and it may be used as a single-atom catalytic material in the future [16,17]. As for the adjustment of the physical properties of γ-GY, e.g., the electronic and magnetic properties, the introduction of impurities and surface defects is an effective method [18,19,20,21]. The doping of metal atom clusters is also a feasible method to adjust the surface activity and electronic properties [22,23,24]. GY is also a promising gas sensing material in many fields. Pristine GY has been shown to interact weakly with most common gas molecules, e.g., CO, CH_4_, CO_2_, NH_3_, and NO, among others [25,26]. Doping with Mn can obviously strengthen the chemical interactions between these molecules and the γ-GY surface [26]. For the gas detection of CO molecules, to improve the weak response, the substitution doping with B and N makes GY more sensitive to CO [27]. For the detection of NH_3_, doping the main group atom, e.g., Si, can significantly reduce the recovery time [28]. The substitution doping of B can also enhance the gas sensing properties for several inorganic, small molecules such as NO and NO_2_, as well as organic molecules [29,30]. The doping of noble atoms has also been shown to significantly enhance the chemical interactions between CO and γ-GY [31]. Based on several theoretical studies of GY as a gas sensing material, pristine GY exhibits weak chemical interactions with most small gas molecules. To enhance the gas sensing properties, introducing an adatom onto the surface is advisable. And also, choosing the appropriate doping approach is essential for the selectivity of GY-based sensing materials.

High voltage equipment has been widely used in power stations, transformer substations, and power distribution stations. To guarantee the operational status of equipment such as transformers using oil-paper insulation or gas-insulated switchgears (GISs) using SF_6_ as an insulating medium, detecting impurity gases using gas sensors has been widely studied. For the detection of SF_6_ decomposition, several 2D materials have been explored, and surface modification including doping transition metal atoms or metal oxides are feasible to enhance the chemical interactions between the SF_6_ decompositions and the sensing materials [32,33,34,35,36,37,38,39,40,41]. Several impurity gases, such as CO, H_2_, C_2_H_2_, CH_4_, C_2_H_6_, and C_2_H_4_, will be generated and dissolve in the oil in the transformer after insulation faults occur. So, a dissolved gas analysis (DGA) method is essential for equipment inspection. In view of this, several materials have been studied to detect these kinds of molecules, including metal oxides [42,43,44,45,46,47] and TMDs [46,47,48]. However, the selectivity and the enhancement of the sensitivity should be further considered. In this regard, due to the high surface activity of γ-GY, and the emerge active sites caused by the introduction of Pd [42,44,45], in this study, we propose a DFT study of four typical dissolved gases in transformer oil (H_2_, CO, C_2_H_2_, and CH_4_) which are adsorbed onto Pd-doped γ-GY. Firstly, the adsorption of one Pd atom on γ-GY was discussed, and the most energetically-favorable structure of Pd-GY was found. Then, the adsorption of gas molecules onto the Pd-GY was investigated and the adsorption energy, electron transfer, and electronic properties were studied in detail. Finally, the different responses to these gases were evaluated. We believe that this study may provide a theoretical basis for graphyne-based nanomaterials to detect dissolved gases in oil, and guidance for the future development of nanomaterial-based gas sensors in many fields.

## 2. Methods

All the adsorption of Pd and gas molecules on γ-GY are calculated in the Dmol^3^ module [49]. The Perdew-Burke-Ernzerhof function (PBE) approach by generalized gradient approximation (GGA) was chosen for correction of the exchange-correlation function [50,51]. The total energy, band structure, and density of states (DOS) were also calculated using the GGA-PBE approximation method. The double numerical plus polarization (DNP) method was considered as the basis set for all the calculations, and the DFT semi-core pseudopotential (DSSP) was selected to handle the electrons, that is, a norm-conserving pseudopotential was applied to calculate the core electrons to improve the calculation efficiency. The long-range interactions derived from the Van der Walls force was calculated using the DFT-D2 method, as proposed by Grimme [52]. A cutoff radius of 4.5 Å was set, and this value remained unchanged in this study. All geometric optimizations between the two steps were carried out using a convergence criterion of 1×10^−5^ Ha for energy convergence, 0.002 Ha/Å for force convergence, and 0.005 Å for displacement convergence. A k-point sample in the Monkhorst-Pack grid of 4×4×1 was used for geometric optimization, while a more accurate k-point was considered in the electronic properties with a Gaussian smearing of 0.005 Ha [53]. The binding energy (*E_ads_*) was calculated using the following equation:

(1) $$E_{bind}=E_{Pd-graphyne}-E_{graphyne}-E_{Pd\;atom}$$

where *E_Pd-graphyne_, E_graphyne_,* and *E_Pd atom_* are the total energy of Pd-graphene, pristine graphyne monolayer, and isolated Pd atom. After the adsorption of one Pd atom, the electron transfer was obtained using the Hirshfeld analysis method [54]:

(2) $$Q=-\int \left(\frac{\rho_{0}\left(r\right)}{\sum \rho_{0}{}^{\prime }\left(r\right)}\right)•\left(\rho \left(r\right)-\sum {\rho_{0}}^{\prime }\left(r\right)\right)dr$$

where *ρ(r)* is the total electron density of the selected structure, and *ρ_0_(r)* represents the electron density of every atom if separated. *∑ρ_0′_(r)* is the sum of *ρ_0_(r)*.

**Figure 1:**
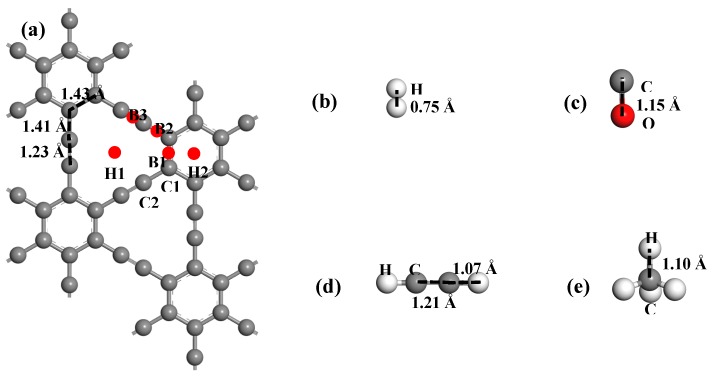
Geometric structures of: (**a**) pristine graphyne with different adsorption sites; (**b**) H_2_; (**c**) CO; (**d**) C_2_H_2_; (**e**) CH_4_.

The adsorption sites of Pd are shown in Figure 1; considering all the structures of the different adsorption sites, the structure with the largest binding energy was chosen for gas adsorption. The adsorption energy of one gas molecule on Pd-graphyne is defined as:

(3) $$E_{ads}=E_{Pd-graphyne/gas}-E_{Pd-graphyne}-E_{gas}$$

where *E_Pd-graphyne/gas_, E_Pd-graphyne_*, and *E_gas_* are the total energy of the structure of the gas molecules adsorbed onto Pd-graphene, Pd-graphyne before the adsorption, and isolation of the gas molecule. All the electron transfers between the gas molecule and Pd-graphyne were also clarified based on the Hirshfeld analysis method. When the electron transfer of Q_T_ is negative, it indicates that the gas molecule extracts electrons from the Pd-graphyne, while if Q_T_ is positive, it indicates that the gas molecule transfers electrons to Pd-graphyne. To gain a deeper understanding of the electron transfer, the electron density difference between the molecule and the Pd-graphyne can be obtained after calculating the electron density as follows:

(4) $$\Delta \rho =\rho_{Pd-graphyne/gas}-\left(\rho_{Pd-graphyne}+\rho_{gas}\right)$$

where *ρ_Pd-graphyne/gas_*, *ρ_Pd-graphyne_* and *ρ_gas_* are the total electron density of Pd-graphyne after adsorbing the molecule, that of Pd-graphyne before adsorption, and that of the isolated molecule, respectively. The purple and red regions are the electron accumulation regions, while the green and blue regions represent electron depletion.

The band structure, the total density of states (TDOS), and the partial density of states (PDOS) were also compared before and after gas adsorption

## 3. Results and Discussion

### 3.1. The Structure of Pd-Graphyne and Dissolved Gas in Transformer Oil

**Figure 2:**
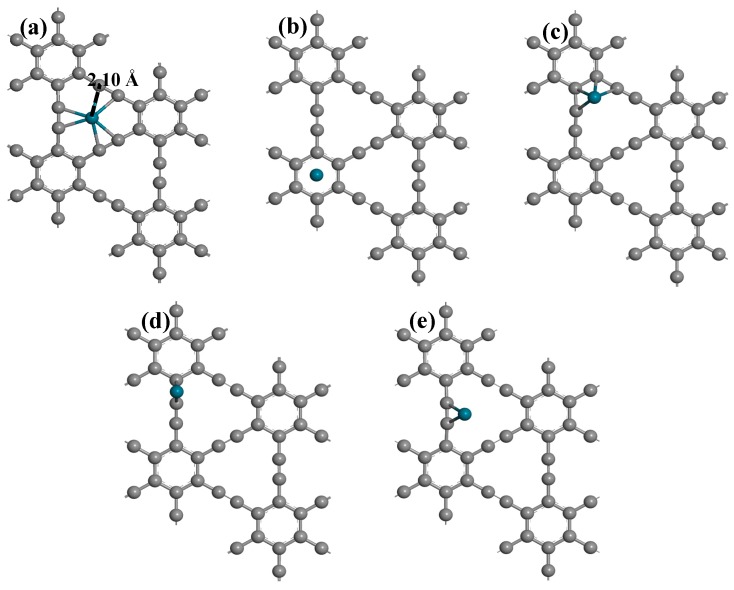
Optimized geometric structures of one Pd atom adsorbed on graphyne: (**a**) H1 site; (**b**) H2 site; (**c**) B1 site; (**d**) B2 site; (**e**) B3 site.

The calculated lattice parameter of γ-GY in this study is 6.89 Å × 6.89 Å, which is consistent with the former results [13,29]. We built a 2 × 2 super cell and put one Pd atom on the surface with different adsorption sites. The stoichiometric proportion of Pd and C is 1:48. The adsorption sites of Pd on the γ-GY surface are shown in Figure 1, including two hollow sites (H1, H2) and three bridge sites (B1, B2, and B3). The initial five structures of Pd-graphyne are fully geometrically optimized. The optimized structures are shown in Figure 2, and the summary of the binding energy and electron transfers are listed in Table 1. According to a comparison of the binding energy, adsorption on the H1 site exhibits the largest energy, indicating that one Pd atom on γ-GY has the most energetically-favorable structure. In Figure 2a, the Pd atom is located in the middle of three C≡C bonds. The distance between Pd and one hybridized C atom is about 2.10 Å. It is important to note that the Pd atom is not totally in the plane of the γ-GY, but rather, it extrudes from the surface by about 0.057 Å. The binding energy of the Pd on the H1 site is −2.45 eV, with an electron transfer from Pd to γ-GY of +0.363 e. The adsorbed Pd atom is positively charged. The adsorption on the B3 site exhibits the second largest adsorption energy, while other adsorption energies are smaller. Based on a comparison of the binding energy, we will focus solely on the structure with the largest binding energy for further discussion, i.e., electronic properties and gas adsorption.

**Figure 3:**
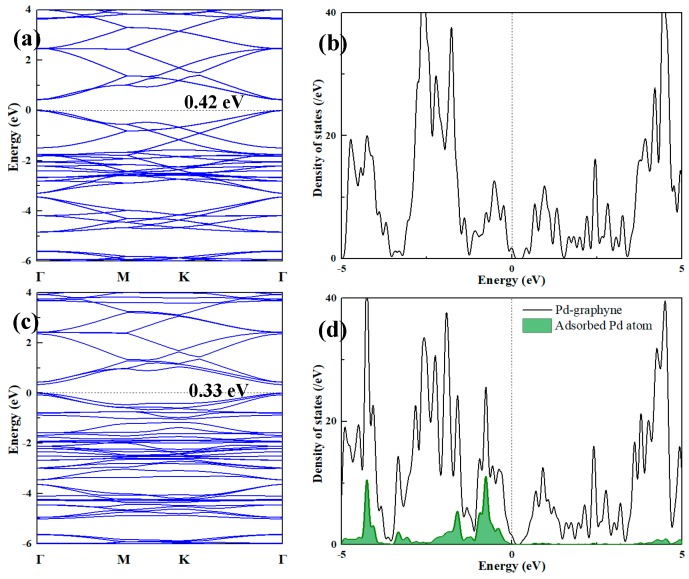
(**a**) Band structure and (**b**) TDOS of pristine graphyne; (**c**) Band structure and (**d**) TDOS of Pd-graphyne and PDOS of adsorbed Pd atom.

The band structures and DOS of pristine γ-GY and Pd-graphyne obtained by the PBE method are shown in Figure 3. Pristine γ-GY has a direct band structure with a 0.42 eV band gap at the Gamma point. This value is very similar to that obtained in other studies (0.43 eV in ref. [23] and 0.435 eV in ref. [55]). The TDOS also shows a gap near 0 eV. It should be noted that the Fermi-level (0 eV) in Dmol^3^ module is set at the highest occupied state, and thus, the imaginary line is in the middle of the highest occupied peak in Figure 3a. So, the non-zero value at 0 eV is mainly because of the broadening of the peak. After doping the Pd atom, the band structure also shows a direct band gap and the value decreases to 0.33 eV. The Pd atom mainly introduces several impurity states near −2.5 eV to 0 eV. The Pd atom also slightly changes its band distribution near 0 eV, and thus, decreases the band gap. We will only consider the adsorption of gas molecules on the structure in Figure 2a in the next section.

The structure of four kinds of gas molecules are shown in Figure 1b–e. H_2_ and CO are diatomic molecules, while the C atoms in C_2_H_2_ are sp hybridized and the C atom in CH_4_ is sp^3^ hybridized. The structures conform to the results obtained in our previous study [46,47,48]. For the initial adsorption structure, we considered two adsorption directions for H_2_ onto the Pd atom (H_2_ parallel to the surface and vertical to the surface), two adsorption directions of CO over the Pd atom (C atom downward and O atom downward), one adsorption direction of C_2_H_2_ (the C≡C bond right above the Pd atom), and two adsorption directions of CH_4_ (one H atom downward and three H atoms downward). Only the structures with the largest adsorption energy of each gas molecule were chosen for further study.

### 3.2. Adsorption of Gas Molecule on Pd-Graphyne

**Figure 4:**
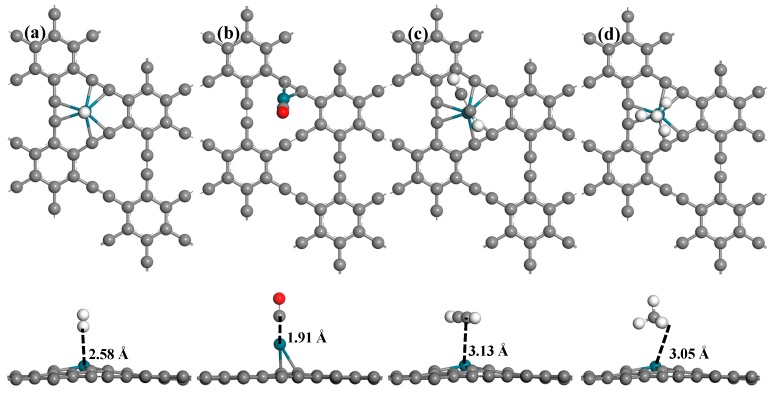
Optimized geometric structures of four kinds of molecule adsorbed on Pd-graphyne: (**a**) H_2_ adsorption; (**b**) CO adsorption; (**c**) C_2_H_2_ adsorption; (**d**) CH_4_ adsorption.

The most energetically-favorable adsorption structures of gas molecules on Pd-graphyne are shown in Figure 4; the adsorption energy and electron transfers are listed in Table 2. The H_2_ molecule prefers to be adsorbed vertically to the surface right above the Pd atom. The adsorption brings only −0.08 eV adsorption energy and −0.059 e electron transfer. The adsorption distance is 2.58 Å. The Pd atom remains its former position. The adsorption of CO occurs somewhat differentely. The C atom in CO prefers to be right above the Pd atom, which moves from the H1 site to the B3 site (between one C≡C bond). The CO obtains −0.080 e from Pd-graphyne, indicating its role as an electron acceptor. The adsorption energy of CO on Pd-graphyne reaches −1.11 eV, which is the largest among these four molecules. The adsorption of C_2_H_2_ exhibits only −0.16 eV adsorption energy, with a very small electron transfer (−0.015 e) compared to other adsorptions. Finally, one sp hybridized C atom is right above the Pd atom. The CH_4_ adsorption has −0.13 eV adsorption energy and −0.063 e electron transfer. For the adsorption of C_2_H_2_ and CH_4_, the adsorption distance is much larger compared to the adsorption of H_2_ and CO, and the position of the Pd atom is unchanged. To sum up, only CO adsorption causes a significant change in the structure of Pd-graphyne, with much larger adsorption energy.

**Figure 5:**
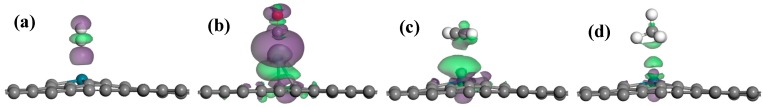
Electron density difference (EDD) of four kinds of molecules adsorbed on Pd-graphyne: (**a**) H_2_ adsorption; (**b**) CO adsorption; (**c**) C_2_H_2_ adsorption; (**d**) CH_4_ adsorption. The purple region denotes the electron accumulation region, while the green region represents electron depletion (the isosurface of (a), (c), and (d) is 0.005 eÅ^−3^; the isosurface of (b) is 0.02 eÅ^−3^).

To perform a more advanced analysis of the interactions, the electron density difference (EDD) was calculated; the results are shown in Figure 5. For the adsorption of H_2_, an obvious electron accumulation occurs near the upper H atom and between the H_2_ and Pd atoms, while the depletion region is between the two H atoms. However, Pd-graphyne does not show any obvious electron density change. The CO adsorption results in an obvious change of the electron distribution. An apparent electron accumulation occurs between the molecule and the Pd atom, while the depletion is around Pd and between C and O atoms. The electrons prefer to move between the molecule and the Pd atom, rather than beneath the Pd. The adsorption of C_2_H_2_ causes obvious electron depletion around Pd, and changes the electron distribution of Pd-graphyne to some extent. As for the adsorption of CH_4_, only a slight depletion region can be found around the Pd atom. Given the different isosurface in Figure 5b, the CO adsorption yields the largest electron redistribution, and the C_2_H_2_ adsorption also causes a considerable change in the electron density of the Pd-graphyne; however, the other two adsorptions only result in a smaller effect. The relationship of electron transfer and electronic properties after adsorption will be discussed in the following section.

### 3.3. Electronic Properties of Pd-Graphyne and Gas Sensing Evaluation

**Figure 6:**
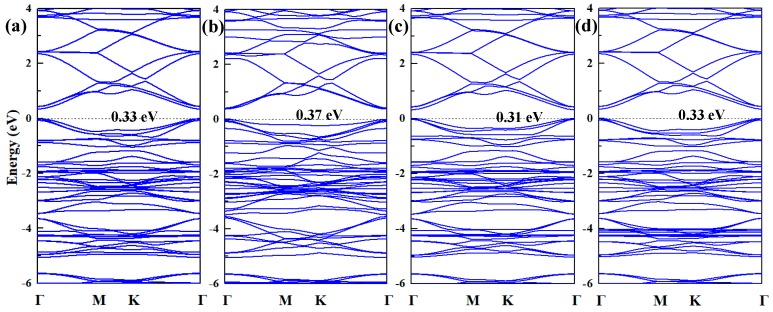
Band structure of (**a**) Pd-graphyne/H_2_; (**b**) Pd-graphyne/CO; (**c**) Pd-graphyne/C_2_H_2_; and (**d**) Pd-graphyne/CH_4_.

To investigate the effect upon the electronic properties of the Pd-graphyne, the band structure and DOS before and after adsorption were compared. The band structures of Pd-graphyne after adsorbing gas molecules are shown in Figure 6. All the band structures still show a direct band gap, but the gap values have some differences. The H_2_ and CH_4_ adsorptions do not exhibit any obvious change of band structure. However, the CO adsorption increases the band gap, while C_2_H_2_ decreases it. Considering the electron distribution in Figure 5, only the adsorptions of CO and C_2_H_2_ yield obvious changes in electron distribution and change the electronic properties of Pd-graphyne. As a result, the band gap changes only after the adsorption of CO and C_2_H_2_.

**Figure 7:**
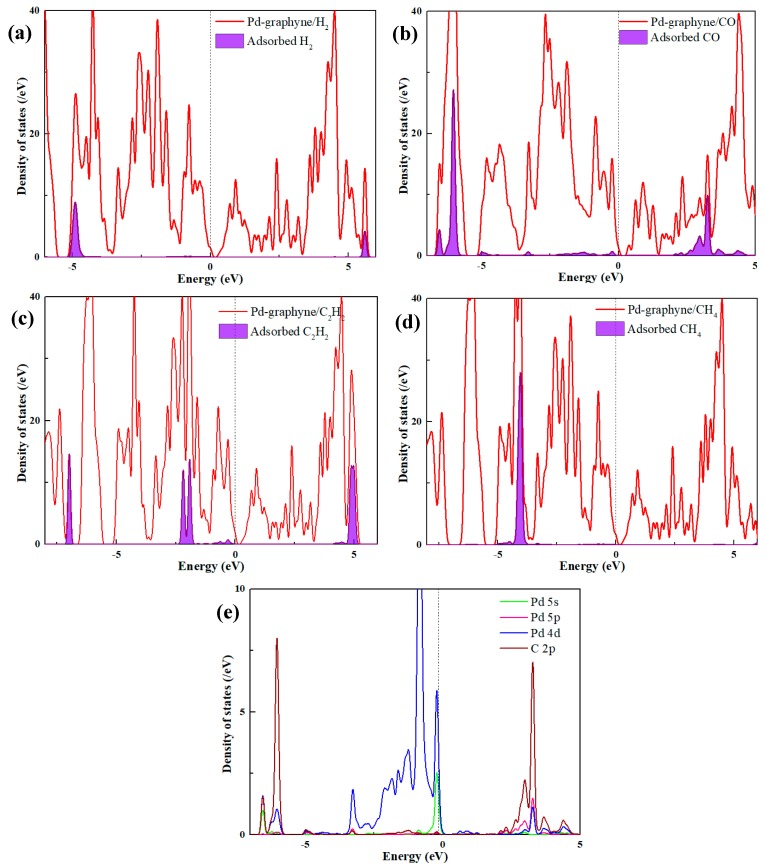
TDOS and PDOS of (**a**) Pd-graphyne/H_2_; (**b**) Pd-graphyne/CO; (**c**) Pd-graphyne/C_2_H_2_; (**d**) Pd-graphyne/CH_4_; and (**e**) atomic orbitals of CO adsorption.

To further investigate the electronic properties, the TDOS of Pd-graphyne after adsorption and the PDOS of the adsorbed molecule are shown in Figure 7. For the adsorption of H_2_ and CH_4_, the states near the Fermi level do not exhibit obvious changes. However, the adsorbed CO or C_2_H_2_ molecule introduces several impurity states just below the Fermi level; thus, these states change the electronic properties of the Pd-graphyne near 0 eV. Furthermore, the new band gap has some differences compared to the Pd-graphyne before adsorption. Because the CO adsorption has a much shorter adsorption distance, larger adsorption energy, and significant changes in structure and electron distribution, the chemical interactions between the Pd atom and CO are explored using the PDOS of the atomic orbitals of the Pd and C atoms in CO, as shown in Figure 7e. The states between −7 eV and −6 eV consist of Pd 5s, Pd 4d, and C 2p orbitals. The states between −3 eV and 0 eV are mainly Pd 4d and 5s orbitals. As for the antibonding orbitals above 0 eV, the states are composed of Pd 4d, 5p, and C 2p orbitals. The obvious hybridizations of peaks near −6 eV and +3 eV between the Pd and C atomic orbitals show significant chemical interactions between these two atoms. In summary, the largest adsorption energy (−1.11 eV), largest electron transfer (−0.080 e) of CO compared to other adsorptions, and the significant state hybridization between the Pd and C atoms provide evidence for the strong interactions between CO and Pd-graphyne.

**Figure 8:**
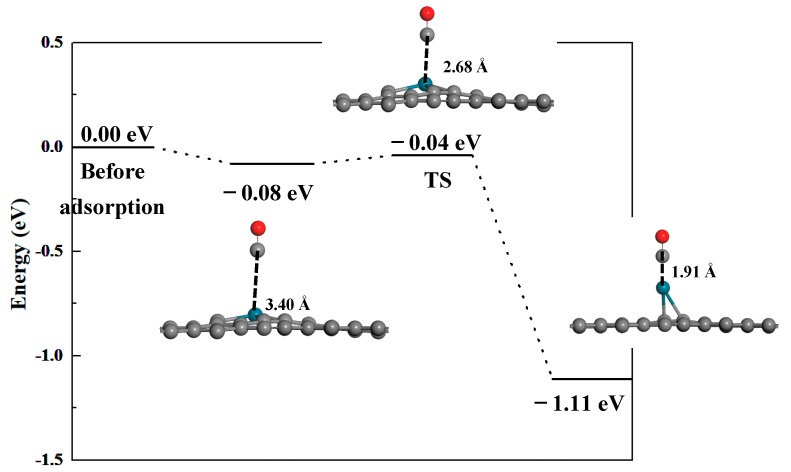
Transition state of CO adsorption on Pd-graphyne.

Resistance type gas sensors have attracted much attention for their gas sensing properties. Electrical conductivity is strongly affected by the band structure of the sensing materials, and the change of the resistance can be evaluated by the band gap [56]:

(5) $$\sigma \propto e^{\left(-E_{g}/2k_{B}T\right)}$$

where *σ* represents the evaluated conductance of the gas sensor, *k_B_* is the Boltzmann constant, and *T* is the working temperature of the gas sensor. It can be seen that in a fixed working temperature, a smaller band gap leads to a larger conductance and vice versa. In this study, the band gap of Pd-graphyne is 0.33 eV, increasing to 0.37 eV when adsorbing CO, while decreasing to 0.31 eV after C_2_H_2_ adsorption. As a result, the conductance rises when detecting C_2_H_2_ and falls when adsorbing CO. However, another point should be also considered: the recovery time of the sensor is an important aspect relative to its the sensing properties. A very short recovery time can promote the desorption process, but rapid desorption may lead to a higher limit of detection. So, the recovery time should be kept within an acceptable range. The recovery time can be evaluated as follows [57]:

(6) $$\tau =A^{-1}e^{\left(-E_{bar}/k_{B}T\right)}$$

where *A* denotes the apparent frequency factor, *k_B_* is the Boltzmann’s constant, and *T* is the working temperature, respectively. *E_bar_* is the energy barrier of the molecule desorption from the surface. For the adsorption of H_2_, CO, and C_2_H_2_, as shown in Figure 4, during the adsorption process, the structure of Pd-graphyne shows nearly no change, and no chemical bonds are broken. As a result, the adsorption does not undergo any endothermic process, and has no energy barrier. Moreover, these three adsorptions mainly show Van der Waals interactions, and no obvious bond formation nor bond breakages have been observed. So, the desorption process of these three gas molecules will not experience a transition state, and the energy barrier of the desorption can be seen as the absolute value of the adsorption energy. The transition state of the adsorption process for CO is shown in Figure 8. It can be seen that the value of the energy barrier of the desorption process is only 1.11−0.04 = 1.07 eV. This value is smaller than the adsorption energy of CO (1.11 eV). As a result, the total energy barrier of desorption is still 1.11 eV, which is the same as that of the adsorption energy. So, for these four adsorptions, the absolute values of *E_bar_* and *E_ads_* are the same. The value of *A* can be evaluated as 10^12^ s^−1^, which is consistent with results published in other studies [58]. According to the above precondition, the desorption times of H_2_, C_2_H_2_, and CH_4_ are 2.25 × 10^−11^ s, 5.08 × 10^−10^ s, and 1.58 × 10^−10^ s, which are much smaller than that of CO, i.e., 5.92 × 10^−6^ s at 298K. At room temperature, CO is hard to desorb from the surface, but much faster desorption of C_2_H_2_ may result in a much higher detection limit. However, if the working temperature reaches 498K, the desorption time of CO decreases to about 0.171 s; this shorter time is suitable for CO sensing. As a result, the Pd-graphyne-based gas sensor may have very good selectivity for CO at higher temperatures.

## 4. Conclusions

This study discussed the structure and electronic properties of Pd-graphyne, as well as its adsorption properties for four common dissolved gases in transformer oil (H_2_, CO, C_2_H_2_, and CH_4_). Pd atoms prefer to be adsorbed on the H1 site with the largest binding energy (−2.45 eV) and electron transfer (+0.363 e). The introduction of a Pd atom decreases the band gap and brings in impurity states just below 0 eV. Among the four types of gases, CO adsorption yields the largest adsorption energy and electron transfer. Moreover, the Pd atom moves from the H1 site to B3 site, resulting in obvious changes in the geometric structure and electronic properties of Pd-graphyne. The band gap becomes larger after CO adsorption, but smaller after C_2_H_2_ adsorption. The strong chemical interactions between Pd-graphyne and adsorbed CO are mainly attributed to the hybridization of the atomic orbitals of the Pd and C in CO. The recovery properties of the Pd-graphyne-based gas sensor show that the recovery time is about 0.171 s for CO desorption at 498K. This acceptable result indicates that this approach may be applied in the realization of a high selectivity gas sensor for CO detection.

## Figures and Tables

**Table 1:** Binding energy and electron transfer of one Pd atom onto a graphyne monolayer.

Adsorption Site	*E_bind_* (eV)	*Q_T_* (e)
H1 site	−2.45	+0.363
H2 site	−1.08	+0.323
B1 site	−1.59	+0.344
B2 site	−1.52	+0.315
B3 site	−1.76	+0.273

**Table 2:** Adsorption energy and electron transfer of H_2_, CO, C_2_H_2_, or CH_4_ adsorbed on Pd-graphyne.

Structure	*E_ads_* (eV)	*Q_T_* (e)
Pd-graphyne/H_2_	−0.08	−0.059
Pd-graphyne/CO	−1.11	−0.080
Pd-graphyne/C_2_H_2_	−0.16	−0.015
Pd-graphyne/CH_4_	−0.13	−0.063
